# Micropatterning of planar metal electrodes by vacuum filling microfluidic channel geometries

**DOI:** 10.1038/s41598-018-32706-6

**Published:** 2018-09-26

**Authors:** Stelios Chatzimichail, Pashiini Supramaniam, Oscar Ces, Ali Salehi-Reyhani

**Affiliations:** 1 Department of Chemistry, Molecular Sciences Research Hub, Imperial College London, London, W12 0BZ UK; 2 Institute of Chemical Biology, Molecular Sciences Research Hub, Imperial College London, London, W12 0BZ UK; 3 fabriCELL, Molecular Sciences Research Hub, Imperial College London, London, W12 0BZ UK

## Abstract

We present a simple, facile method to micropattern planar metal electrodes defined by the geometry of a microfluidic channel network template. By introducing aqueous solutions of metal into reversibly adhered PDMS devices by desiccation instead of flow, we are able to produce difficult to pattern “dead end” or discontinuous features with ease. We characterize electrodes fabricated using this method and perform electrical lysis of mammalian cancer cells and demonstrate their use as part of an antibody capture assay for GFP. Cell lysis in microwell arrays is achieved using the electrodes and the protein released is detected using an antibody microarray. We show how the template channels used as part of the workflow for patterning the electrodes may be produced using photolithography-free methods, such as laser micromachining and PDMS master moulding, and demonstrate how the use of an immiscible phase may be employed to create electrode spacings on the order of 25–50 μm, that overcome the current resolution limits of such methods. This work demonstrates how the rapid prototyping of electrodes for use in total analysis systems can be achieved on the bench with little or no need for centralized facilities.

## Introduction

Microfluidics have been established as an essential analytical platform in chemistry. A critical bottleneck to even wider adoption of microfluidics is the significant barriers to entry which remain, notably complexity, cost and access to centralised facilities. However, increasing demand is driving the development of low-cost microfluidic tools and fabrication methods. Historically, microfluidic devices have been fabricated from silicon or glass by wet-etching and photolithography in cleanroom environments^1,2^. The advent of soft lithography and the use of polydimethylsiloxane (PDMS) was a significant stride toward simplified fabrication. Certainly, much of soft lithography’s success is owed to the biocompatibility and optical properties of PDMS^3^, although in certain cases care must be taken^4,5^. Alternative processes are being developed that are able to fabricate channels and features directly and do not rely on wet etching, such as 3D printing^6–8^, hot embossing and injection moulding^9–11^, CNC (computer numerical control) machining^12^, and laser based approaches^13^. While there is considerable promise in these methods, currently, start-up costs remain high, materials are limited and concerns regarding surface quality and feature resolution have yet to be fully addressed^12^.

A variety of on-chip features and strategies have been reported for bioanalytical applications by enabling on-chip manipulation and analyses of biologically relevant fluids and objects, such as cells. These approaches may be optical^14^, electrical^15^, magnetic^16^ or acoustic^17^ in addition to hydrodynamic and valve based approaches from the geometry inherent to the design of the channels themselves^18,19^. For electric-based approaches, electrodes may be incorporated by either inserting traditional electrodes/wires into a device or by patterned deposition of metal on a glass substrate which supports the device.

Electrodes on-chip have been used to achieve electroosmotic flow^20^, electrophoresis^11^, dielectrophoresis^15^, electrical lysis^21^ and based on these principles have been employed as electrochemical sensors, sorters and pumps.

Conventional fabrication involves depositing metal through either physical vapour deposition, most commonly by sputtering or thermal evaporation in addition to e-beam evaporation and electrodeposition^20^. Depending on the metal, patterning may be achieved by lift-off, wet-etching or dry-etching. Although these techniques are able to produce high resolution features with precise layer thickness and uniformity, they are complex, require expensive specialised equipment and access to cleanroom facilities. Microelectrodes have been key to the analytical capabilities of microfluidic devices^22,23^ and as such efforts to simplify their fabrication amenable to low-cost rapid prototyping are also being made^24^.

One substitute for patterned metal electrodes is to fill dedicated microfluidic electrode channels with a conductive liquid^25–27^. The electrode channels may be fabricated using soft-lithography as per regular channels or even form part of the same fluidic channel separated by microposts^28^ thereby simplifying the entire fabrication process while allowing micron-scale features to be achieved with no additional alignment steps required. Despite the benefits of 3D electrodes, such as field uniformity, planar electrodes are more easily integrated into microfluidic devices since channel design and placement is much less of an issue.

Alternative methods of patterning planar electrodes include printing^29,30^, using conductive ink pens^31,32^ and templating or micromoulding^33^. Wu *et al*. developed an inkjet-printing method to pattern microelectrodes directly on PDMS using silver nanoparticles^34^. A three-electrode electrochemical sensor was fabricated and used as a glucose biosensing system. Microcontact printing is a technique often used for surface patterning of biomolecules, where an elastomeric stamp transfers a surface-reactive reagent, or ink, to a substrate to produce 2D patterns^14^. Microcontact printing has been used to chemically pattern a surface onto which nanoparticles selectively adhere and form a seed layer onto which conductive films are established^35,36^. However, as with microcontact printing in general, diffusion and spreading of the ink and stamp deformation can affect the resolution and reproducibility of the printed pattern.

Ebina *et al*. deposited a thin metal layer using wet chemistry, deposited by Tollens’ reaction^37^. Patterns were produced by spin-coating positive photoresist onto the metal layer and subsequent photolithography using a mask which is required each time a device is produced. Despite the approach being potentially time-consuming it enables the fabrication of micron-scale features. Hong *et al*. patterned silver electrodes by the Tollens’ reaction using the fluidic channels of a PDMS template^38^. Although, this requires the additional step of producing fluidic channels, it reduces the reliance on photolithography to only the fabrication of the reusable silicon master from which the PDMS template is made.

Here we describe a facile, rapid and low-cost approach to planar microelectrode fabrication using the fluidic channels of a reusable PDMS template to deposit and pattern electrodes using wet chemistry. We use a channel outgas technique whereby the chip is exposed to a vacuum within a desiccator to remove air from the microfluidic channels which is replaced with electroless plating solution upon restoration of atmospheric pressure^39^. With this method we are able to easily fill complex channel designs as well as single entry, internally terminated channels, enabling the fabrication of interdigitated electrodes and non-continuous branched microstructures, inaccessible to previously reported techniques that rely on flowing reagent through microchannels. As our method is not reliant on syringe pumps to fill templating channels it has the added benefit of avoiding inadvertent fouling of tubing and syringes by any metal plating solution.

We demonstrate the functionality of the microelectrodes by performing cell lysis in a microwell device suitable for single cell analysis and report optimal conditions for lysis. We further develop the technique to use a non-lithographically produced master to produce microchannels to template electrodes. We demonstrate how the current resolution limits of such methods may be overcome to achieve planar microelectrode separations on the order of tens of microns by using an immiscible oil phase to separate aqueous plating solution in the fluidic channels.

## Results

### Assessing fabrication parameters governing electrode quality

The electroless deposition of silver in microfluidic channels offers significant advantages over conventional fabrication methods. It is fast, scalable and it allows the electrode layer to be incorporated into the device both before and after the incorporation of the mechanical components such as the microfluidic channels. For that matter, the mass-transport kinetics of the electroless deposition are of paramount importance. Given the inherent mass-transport restrictions in microfluidic channels as opposed to batch-like processes, one needs to consider the effect of the plating bath concentration.

To do this, the conductivity of the silver thin films was measured for a range of initial silver ion concentrations filling the microfluidic channels. The effect of temperature on the plating of electrodes was also examined (see Supplementary Information; Figure S1); however strongly adherent electrodes suitable for subsequent applications were only produced when a hot plate temperature of 100 °C was used.

**Figure 1:**
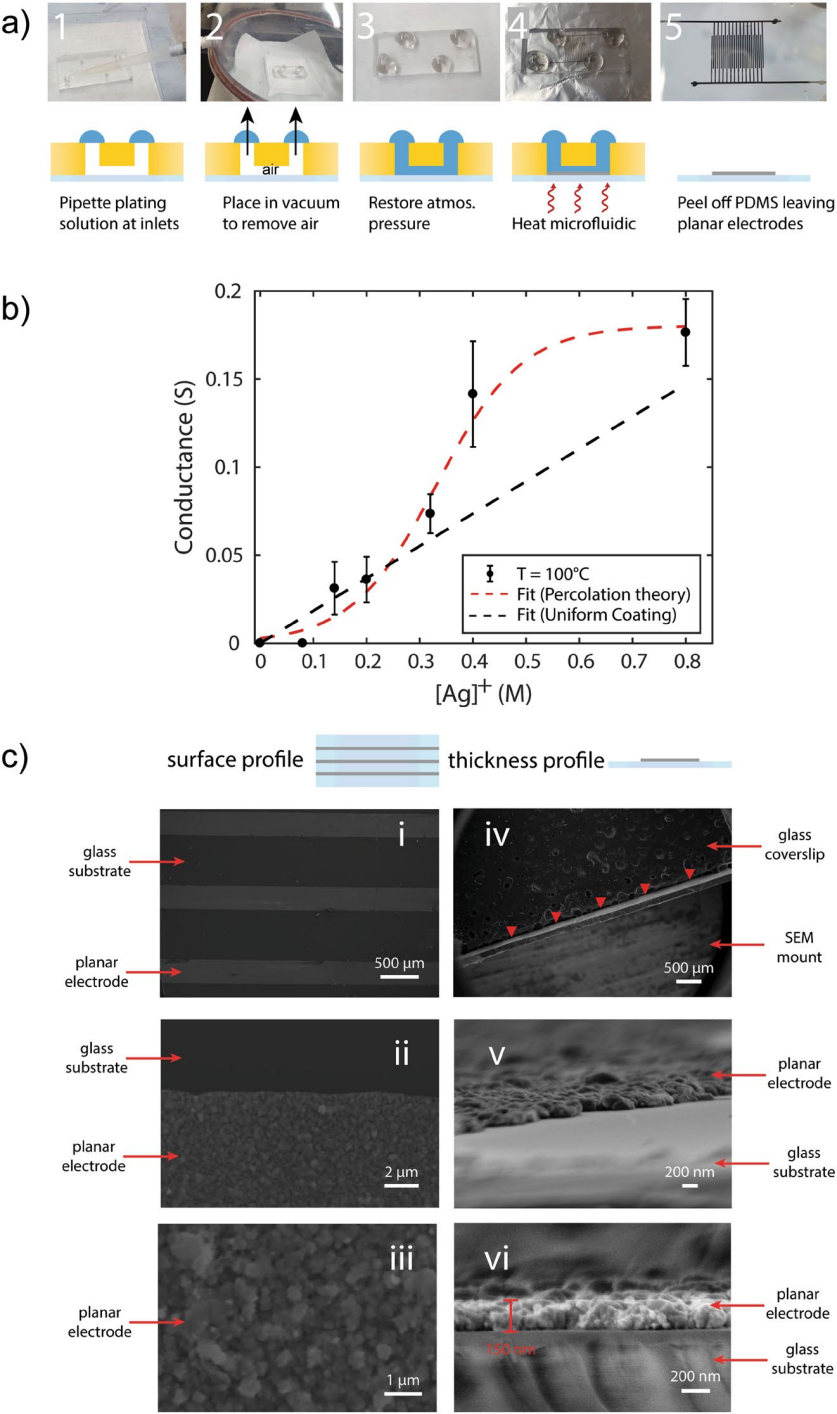
Deposition and characterisation of electrodes. (**a**) Illustration of the plating process: 1, a PDMS mould is drilled with inlet holes and reversibly bonded to a glass coverslip or slide; 2, plating solution is pipetted so as to completely cover the inlets; 3, the microfluidic device is placed in a desiccator and allowed to degas for 5 minutes; 4, atmospheric pressure is restored allowing the plating solution to fill the vacuum in the main and side-channels; 5, the device is heated on a hotplate for 5 minutes at 100 °C; and 6, the PDMS mould is detached from the glass slide leaving planar electrodes. Shown are interdigitated electrodes (width = 300 μm, spacing = 200 μm). Scale bar corresponds to a length of 10 mm. (**b**) To determine the functionality of the electrodes, conductivity of main channel electrode is measured. Conductance varies as a function of the initial silver ion precursor concentration and shown are the results using electrodes cured at 100 °C. The dashed black line is an estimate of conductance when making the uniform coating assumption, which would apply in the case of non-porous films. The dashed red line is a Boltzmann fit to the data (R^2^ = 0.97) suggesting that electrode formation is in line with percolation theory. (**c**) SEM analysis of the planar electrodes produced on glass coverslips. i-iii: Images show the surface profile of the electrodes at increasing magnification. iv-vi: Images show the lateral profile of the electrodes to determine electrode thickness. The red arrow heads in iv indicate the location of the planar electrodes.

Figure 1b shows how the conductance of the electrodes varies with increasing silver ion concentration in the plating solution. Under the assumption of uniform coating of the glass substrate, one would expect the thickness of the metallic films to scale proportionally to the concentration of the silver present in solution. For rectangular channels undergoing uniform coating, the silver film thickness, *h*_*Ag*_, would vary according to ${h}_{Ag}=\frac{{V}_{chip}}{\,{S}_{chip}\,}\frac{{M}_{A{g}^{+}}{C}_{A{g}^{+}}}{{D}_{Ag}}$, where *V*_*chip*_ and *S*_*chip*_ are the volume and surface area of the microfluidic channels respectively, *M*_*Ag*+_ and *C*_*Ag*+_ are the molecular weight and concentration of the silver complex respectively and *D*_*Ag*_ is the density of metallic silver. As such, the conductance of the film, *G*_*film*_, would be expected to vary according to ${G}_{film}=\frac{{h}_{Ag}W}{\rho \,L}$, where *W* is the width of the film, *ρ* is the resistivity of silver and *L* is the conductive path length of the electrode. It is evident from Fig. 1b that the conductance of the films produced in our devices does not scale linearly as would be the case for uniform coating. The behaviour of this is attributed to the observation by others that chemically grown silver films deposit in island clusters^40^ and a linear relationship between film thickness and conductance across a film is not typically observed. This type of deposition behaviour is described by classical percolation theory. Indeed, we find that the conductance of the devices obeys the sigmoidal-Boltzmann model (R^2^ = 0.97), a model commonly employed to simulate electrical conductivity percolation parameters^41^. A percolation model would capture the lack of any conductivity at the lower concentration regime, attributing the discrepancy to the larger porosity of the films formed at lower concentrations^42^. We note that, in addition to other sources of variation, the limitation of the two-point probe technique, whereby the probe wire resistance is no longer negligible compared to the microelectrodes, can contribute to the higher variation in measured electrode conductance for the higher conductance films.

The topography and geometry of the planar microelectrodes were characterised by scanning electron and optical microscopy. For scanning electron microscopy (SEM), a diamond-tipped scriber was used to cut out 10 mm wide sections of coverslips supporting interdigitated electrodes. These were mounted on SEM specimen stubs to profile either the surface (Fig. 1c,i–iii) or thickness (Fig. 1c,iv–vi) of the electrodes. Here, since only areas within the microchannel are exposed, the edges of the electrodes are distinct and well-defined (Fig. 1c,ii). The PDMS protects unexposed areas of the substrate and as such there is no background density of particles. Their metal-like appearance, i.e. reflective and opaque, suggests that there is a complete coverage of silver in the exposed regions. The morphology of the electrodes at high magnification (Fig. 1c,iii) shows a percolated state and that complete coverage has, indeed, been achieved. Devices were also imaged optically during the deposition process and it was determined there was minimal to no lateral shrinkage (Figure S2), demonstrating that the method reproduces the microchannel geometry with high fidelity. Samples were also mounted at 90° to determine their thickness profile. The thickness of the electrodes was measured to be ~150 nm (Fig. 1c,vi).

### Application to electrical lysis of cells

The electrodes were developed to achieve electrical lysis of human cancer cells. A PDMS chip made of a 14 × 9 array (n = 126) of cylindrical microwells was used to capture cells for lysis; each well was 150 μm in radius and 35 μm in depth, enclosing a 3.5 nL volume when sealed. Microwell arrays optimized for the capture of single cells, typically, have diameters on the order of that of the cell but is not the case here. To generate electric fields within the chamber, a portion of the electrodes must be in contact with the fluid in each chamber and so sufficient surface area must be provided in each well, hence their size.

**Figure 2:**
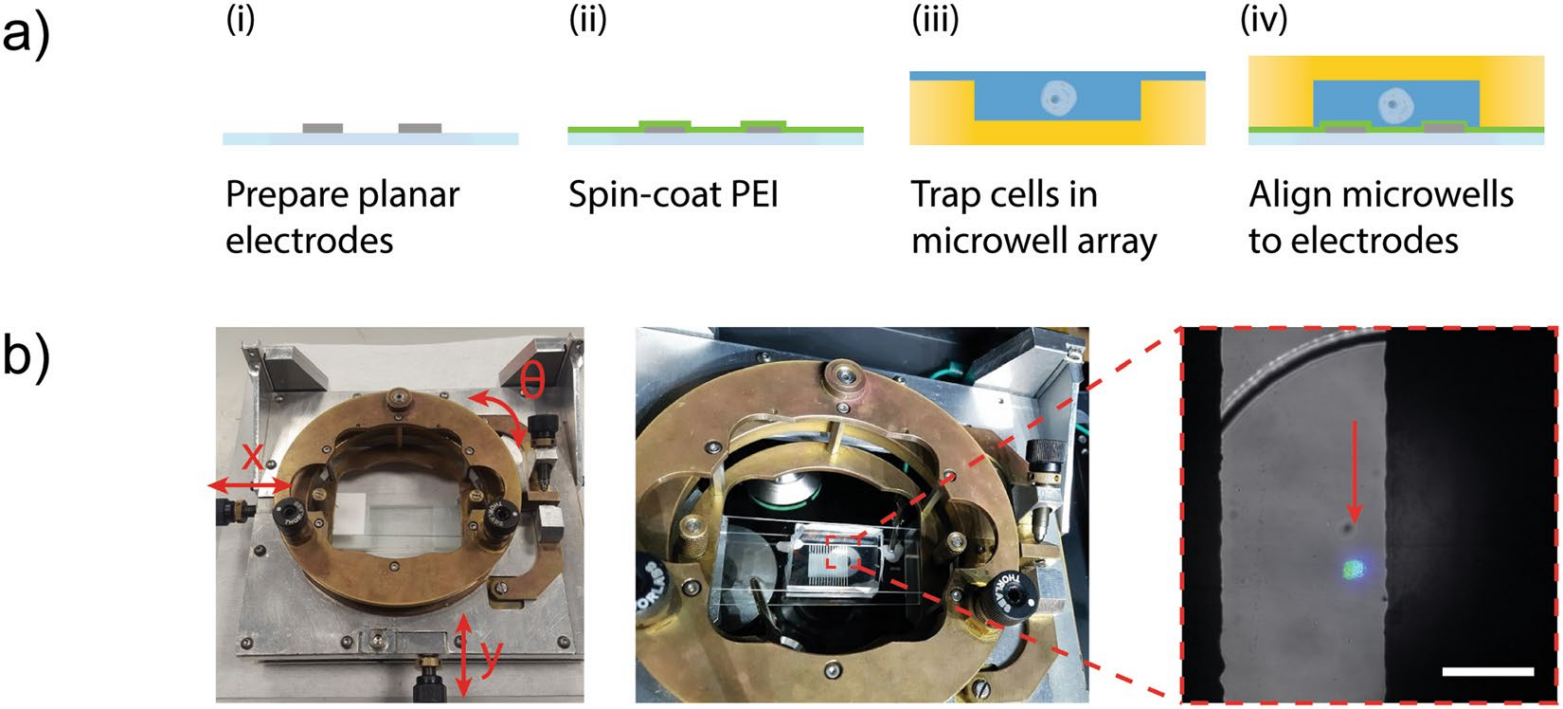
Microwell arrays and patterned electrodes. (**a**) Microelectrodes with an interdigitated geometry are fabricated using the vacuum filling method. A layer of the polymer PEI is spun coat onto the electrodes to promote adhesion and prevent lift-off coating (1% w/v, 2500 rpm). A microwell array (14 × 9; well dimensions: 300 μm diameter, 50 μm height) is air-plasma treated and wetted with buffer solution before sedimenting a solution of cells. The microwell array containing cells is aligned to the interdigitated microelectrode. (**b**) A custom-built XYZ stage aids alignment. The inset composite image shows a nuclear stained (blue), GFP-expressing (green) cell trapped in a single microwell between two microelectrodes. Scale bar 100 μm.

To perform lysis experiments, microwell arrays containing cells are aligned to microelectrode coverslips (Fig. 2a). The microwell array is first treated with an air plasma for 1 minute to render the surface hydrophilic and ensure fluid fills the microwells without resulting in any air bubbles. A 4% PBSA solution is added on the well array to pre-wet the chambers and a glass coverslip is used to remove excess solution by scraping. A solution containing a suspension of cells is pipetted to remove any cells not sedimented within the microwells before excess solution is, again, removed by scraping. The microwell array is mounted on a custom-made alignment jig which helps in accurately aligning the wells to the microelectrode coverslip (Fig. 2b).

Under certain electrical conditions necessary for the lysis of cells, we witness deterioration and lift off of portions of the electrodes from the glass substrate. Polyethylenimine (PEI) is commonly used to increase the adhesion of electrode materials^43^. PEI spun coat onto electrodes was able to prevent lift-off and is used for all subsequent experiments.

It is known that a constant applied DC voltage can result in electrolysis and bubble formation on the electrode edges. Electrolysis leads to the formation of hydroxide ions and the reduced volume of the microwells employed here can easily result in an equilibrium concentration of OH^−^ that may exceed the necessary lytic concentration^21^. Although, lysis may be achieved in this manner it is not desirable in maintaining the functionality of proteins released from the cell. Therefore, to minimize hydroxide ion generation and ensure lysis is achieved through irreversible electroporation, an alternating voltage (V_AC_) was applied with a small DC offset (V_DC_). Additionally, continuous oxidation is deleterious to the electrodes and so this helps to prolong their operational lifetime.

The cell occupancy per microwell follows a Poisson distribution (see Supplementary Information; Figure S3) and a cell concentration (0.03 cells/nL) which minimized the occurrence of multiple cells and maximized the occurrence of single cells per microwells was chosen.

**Figure 3:**
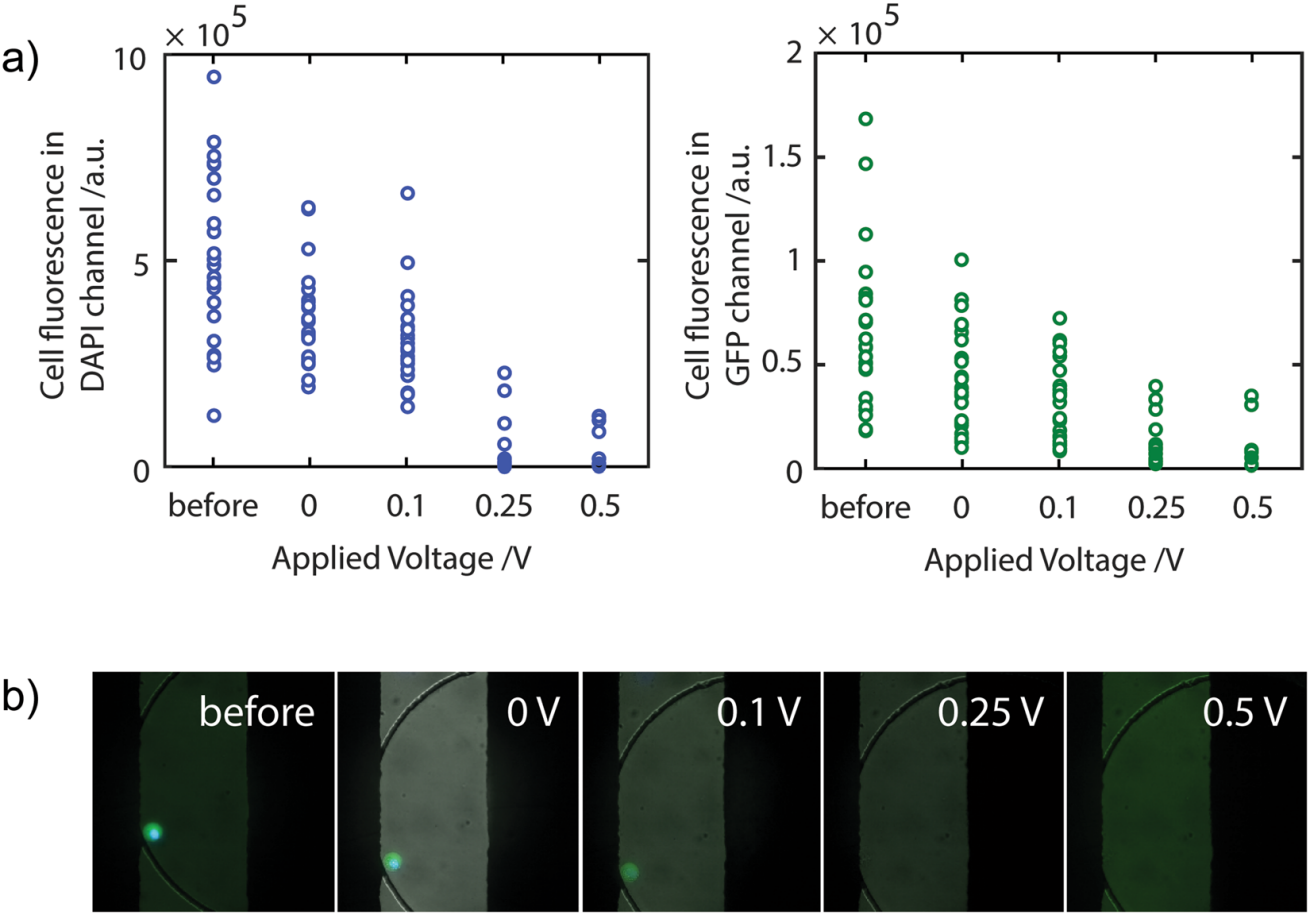
Electrical lysis of cells. (**a**) Using the electrodes, an electric field is applied to the cells within the microwells. An alternating voltage V_ac_ = 10 V_pp_ 1 MHz, superimposed to a constant DC voltage, V_DC_ was applied. The GFP (left plot) and nuclear stain (right plot) fluorescence signal per cell was measured after 5 minute application of the electric field. (**b**) Combined bright field/GFP/H33342 images showing the effects of the electrical field on a cell at each of the DC voltage offsets indicated.

To determine the optimal conditions to induce complete lysis of the MCF7-GFP cells, a range of applied voltages and frequencies were tested. While relatively low strengths of the applied electric field are expected to induce reversible electroporation, irreversible electroporation leading to cellular lysis only occurs when the critical electric field strength is exceeded^44^. This is also reflected in our results **(**Fig. 3a**)** where the degree of lysis may be assessed by the measuring the integrated fluorescence of cytoplasmic GFP or nuclear dye. An initial reduction of the cytosolic GFP and nuclear dye fluorescence was observed upon application of a small DC offset (V_DC_) to the applied voltage. However, only at V_DC_ voltages higher than 0.25 V did we observe complete loss of fluorescence signal accompanied with cellular membrane disintegration (Fig. 3b).

Varying the frequency of V_ac_ was observed to have a minimal effect on the efficiency of lysis. As such, higher frequencies were typically favored as they were found to prolong the operational lifetime of the electrodes.

### Use of electrodes as part of a protein assay

To further demonstrate the utility of the electrodes, we performed an antibody-based protein assay within the microwells to detect protein liberated from electrically lysed cells.

To detect cellular GFP protein, anti-GFP antibody was microarrayed onto PEI-coated electrode-templated coverslips such that antibody spots were centred between the interdigitated electrodes. A suspension of BE cells stably expressing cytosolic GFP and nuclear stained with Hoechst 33342 were loaded into a microwell array and the chip was subsequently aligned to the electrode coverslip. The cell occupancy per microwell was counted and although the electrodes covered a significant proportion of the microwell area cells, that would be otherwise obscured, could be visualised by their fluorescent nuclear stain while increasing the gain of the camera.

An electric field (V_AC_ = 10 V_pp_, V_DC_ = 0.5 V, f = 1 MHz) was applied for 5 min to ensure complete lysis of the cells including the nucleus, which was confirmed by microscopy. Once cells are electrically lysed their intracellular content is able to diffuse into the chamber and the cellular GFP protein freely diffuses and is able to bind to the antibody spot. The array of antibody spots was imaged by TIRF microscopy prior to lysis and 90 min after the application of the electric field, long enough to achieve thermodynamic equilibrium of binding.

**Figure 4:**
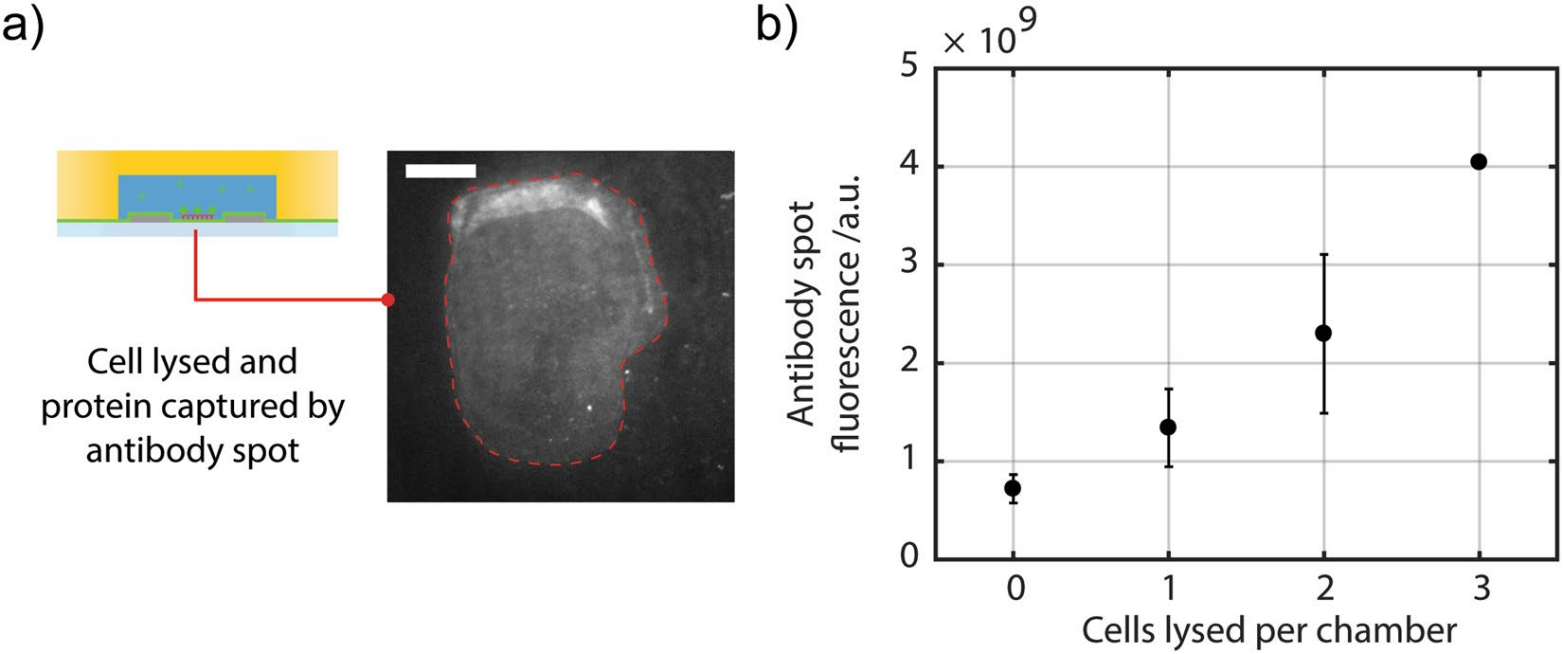
Using microelectrodes in microwells to assay protein from cells. (**a**) The image, acquired by TIRF, shows an anti-GFP antibody spot printed onto a PEI- and microelectrode-coated coverslip and aligned into an individual well of the microwell array. Image shows GFP captured by the antibody spot (within dotted red line). Scale bar length is 25 μm. (**b**) The number of cells per well follows a Poisson distribution. The antibody spot in each microwell captures fluorescent GFP protein from the electrically lysed cells. The results show that, as expected, the GFP signal increases with increasing GFP-expressing cell occupancy.

Figure 4 shows the results of the GFP protein pulldowns within the microwell arrays. A fairly linear response in on-spot fluorescence signal due to the presence of bound GFP protein was obtained with increasing cell occupancy of the chambers (Fig. 4b).

While PEI helps to overcome issues with electrode adhesion, it is not an ideal surface to support an antibody microarray for TIRF microscopy using the wavelengths reported here, owing to the relatively high autofluorescence observed and the degree to which non-specific binding may occur. Additionally, for some electrodes, we observe an increase in fluorescence on their surface post-lysis, which we presume may be due to non-specific binding of GFP released from any cells within the microwell. All these factors would contribute towards underestimating the GFP content of the cells under investigation and thereby reducing the sensitivity of the assay.

Despite the sub-optimal surface conditions, we are able to assay GFP protein within these cells upon lysis using the electrodes produced here. Further work is needed in order to optimize the surface in its capacity to fully support both the electrodes as well as an antibody microarray.

### Control of electrode spacing using an immiscible phase

This method of electrode fabrication does not rely on microelectrode fabrication facilities for the applications demonstrated here; however, as reported we relied on photolithography to produce the SU-8 master from which the PDMS microchannel templates for the interdigitated electrodes were made. The potential of laser micromachining acrylic templates to replace the photo-lithographically produced master.

**Figure 5:**
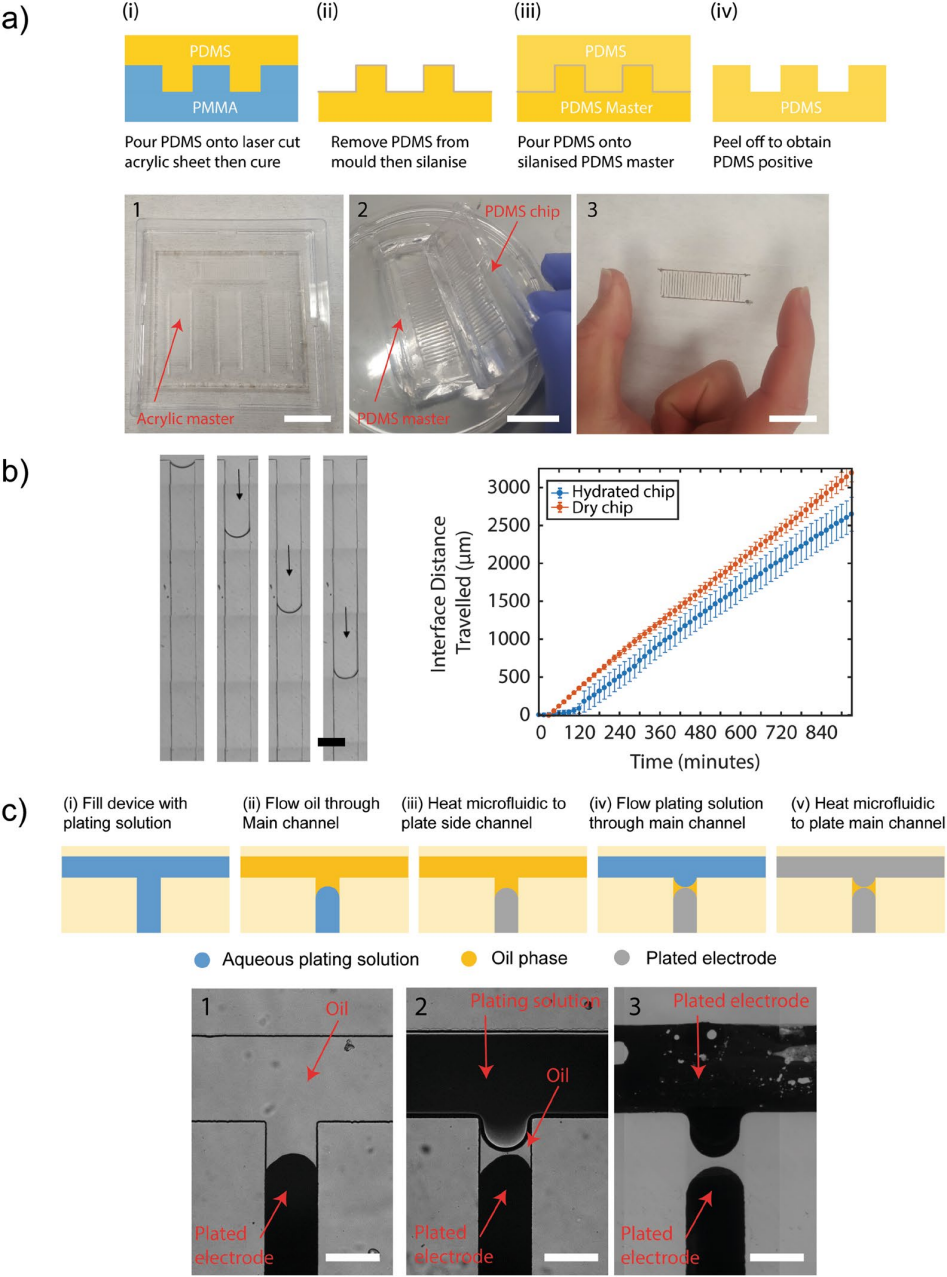
Photolithography free methods of master production and electrode structuring using an immiscible phase. (**a**) A PDMS master (2) is created from a laser micromachined acrylic sheet (1) and used to produce PDMS channels to pattern microelectrodes (3). First, PDMS is poured onto a laser cut acrylic master 1/(i). A PDMS master is produced by (ii) removing the PDMS from the acrylic master and silanising it. (iii) The silanised PDMS serves as a PDMS master upon which more PMDS is poured. Finally, cured PDMS is peeled off the PDMS master 3/(iv) and used to produced microelectrodes. Scale bars are 20 mm. (**b**) On-chip evaporation is a known phenomenon whereby the solution filling the channel can diffuse into the PDMS over time due to its porosity. This results (left image, scale bar 300 μm) in the slow creeping over time of an oil-water interface along each ‘dead-end’ channel which forms the interdigitated electrodes. Graph shows results from images of the chip that were automatically acquired to measure the creeping rate of the interface for both an untreated (dry) chip and a chip that had been immersed in water for 24 hours (hydrated). (**c**) These were exploited to pattern microelectrode pairs with separation lengths much lower than that achievable by the channel geometry alone. Channels were (i) filled with plating solution by vacuum and (ii) the plating solution in the main channel was replaced with mineral oil then (1)/(iii) heat cured to form electrodes. 2/(iv) The oil in the main channel was replaced by fresh plating solution and remained separated from the plating solution in the channels by the immiscible oil phase and produced spatially separated electrodes (3)/(v). Scale bars are 300 μm.

We used a PDMS-moulding technique assisted by laser micromachining microchannel patterns using acrylic sheets. The electrode geometry (Fig. 5a) was cut out of a 1 mm acrylic sheet backed with double-sided adhesive (3 M). The acrylic was adhered to the bottom of a clear plastic petri-dish and PDMS pre-polymer is poured on to the template, degassed and cured. The PDMS is peeled from the acrylic, inverted and placed in another petri-dish and silanised with PFOCTS. The silanised PDMS serves as a negative master mould from which PDMS prepolymer could be poured, cured and easily detached forming microchannel devices that enable the electroless deposition of electrodes (Fig. 5a). This PDMS master moulding method was found to yield reproducible geometries for up to 5 curing cycles before resilanisation or a new PDMS master was required.

The minimum channel width and the separation distance between channels is dictated by the focal spot size of the laser and the scanning resolution of the system used to micromachine the initial acrylic template. Lateral features below a hundred microns with smooth sidewalls are difficult to achieve. However, the electrode spacing may not necessarily need be dictated by the channel geometry directly. For example, Guler *et al*. used hydrodynamic focusing of an etching solution stream to etch a microwire placed across a 200 µm fluidic channel^45^. They were able to precisely control the electrode spacing down to 20 µm by varying the focused widths of the etchant solution.

For the case of cell lysis, reducing electrode separation is advantageous in permitting lower electric field strengths to be used reducing joule heating in operation and prolonging the lifetime of the electrodes. To achieve electrode separations smaller than that of the channel geometry, we explored the use of an immiscible oil phase to separate the aqueous phase of the plating solution (Fig. 5b). As before, the chip was initially filled with plating solution by vacuum filling using a desiccator. Mineral oil was then drawn through the main channel using a syringe (Fig. 5b). The effects of on-chip evaporation cause the oil/water interface to slowly creep along the perpendicular ‘dead-end’ microchannels. The oil in the main channel may be replaced by drawing through more plating solution (Fig. 5c) which remains separated from the vacuum filled plating solution by the oil that remains. It was found that the average creeping rate of the oil/water interface over 15 hours (PDMS thickness = 3 mm) was 3.29 ± 0.13 μm min^−1^ (Fig. 5b). Saturating the PDMS by immersing overnight in water prior to use reduced the average creeping rate to 2.86 ± 0.11 μm min^−1^. These were calculated from large-scale images acquired over time using an automated microscope. Although potentially time consuming, the slow creeping rate allows for considerable control over the spacing of electrode pairs. Using this method (Fig. 5c**)** and waiting 10 min after the introduction of the oil to introduce the second plating solution, electrode pairs with spacings of 36.5 ± 7.9 μm were achieved in this way.

## Discussion

We have demonstrated a simple, facile and rapid method to fabricate planar electrodes for use in microfluidic devices. By utilising the method of desiccation to perform vacuum filling of microfluidic channels planar electrodes were templated onto glass coverslips that are compatible with surface mounted microfluidic devices. The method allows the fabrication of planar microelectrode patterns that are difficult to achieve or unattainable by flow-based approaches. Crucially, we have comprehensively demonstrated the functionality and utility of the electrodes for biological and biochemical studies in performing electrical lysis of human cancer cells in microwell arrays. By incorporating an antibody array, we were able to extend our work to demonstrate protein pulldowns of GFP from electrically lysed cells. While single-cell resolution could be elusive for lower protein-expression targets than the one we chose, it is envisaged that further improvement in the method can be drawn by investigating surfaces other than PEI. Ideally the surface chosen would maintain the mechanical stability of the electrodes when employing conditions that achieve lysis, whilst improving the capability in supporting a sensitive antibody microarray. We chose silver to deposit as electrodes because of its relatively high intrinsic electrical conductance; however, other conducting substances and supporting substrates including plastics may be used. Adhesion of gold electrodes to glass substrates Certainly, it is not beyond the scope of the method to use the PDMS microchannels to prime substrates to functionalise their surface or deposit adhesion promoters which can support subsequently deposited layers.

A limitation of the current method is in patterning discontinuous features. Techniques such as micro-contact printing^46^ are used to pattern substrates by way of transient contact; however, despite their versatility and low cost, it suffers from inhomogeneity of transferred material. One possible solution to this would be to produce a multilayer templating device comprising of a stencil layer, to expose discontinuous regions of the substrate, connected to a second layer comprised of microchannels through which plating solution would flow.

In an endeavour to further simplify and achieve clean-room free methods of operation, we’ve demonstrated that laser micromachining and PDMS master moulding is capable of producing competent electrodes negating the need for photolithography. Furthermore, by exploiting on-chip evaporation and an immiscible oil phase, we could achieve electrode pair spacings significantly lower than that of the channel to channel spacings of the electrode chip design. This has potential in aiding the fabrication of electrodes with chips produced by laser micromachining such that electrode spacings lower than the current spatial resolution limits associated with laser micromachining can be achieved. This is to our knowledge the first time oil-water interfaces have been employed as a structuring tool for electroless plating.

## Methods

### Fabrication of PDMS devices

Microfluidic PDMS chips were prepared using standard soft lithography techniques (electrode templates and microwell arrays) or by a laser cutting-assisted PDMS moulding technique (electrode templates). A device comprising of a main channel (500 μm × 37,5 mm) and 25 side channels (300 μm × 14.5 mm) with a channel height of 50 µm was used for method development. Two 300 µm access holes were drilled (Diama, UK) and 250 µL solution reservoirs were formed by contact bonding cut outs of PDMS. It is necessary that coverslips be subsequently delaminated of the PDMS and so microchannels are sealed by contact bonding alone. To clean and prepare the glass coverslips, they were immersed in an acetone solution and ultra-sonicated for 30 minutes. Following this, the substrate was then washed first with 2-propanol and dried under a stream of N_2_. Subsequently, the glass coverslips were washed with DI water and immersed in a solution of KOH 0.1 M and ultrasonicated for a further 30 minutes. Finally, the slides were washed with DI water, dried under N_2_, immersed in ethanol and ultrasonicated for 30 minutes. The substrates were then dried under N_2_ and contact-bonded reversibly to the PDMS microchannels. This protocol was adopted from methods to prepare coverslips for antibody microarrays; it is possible to simplify this and reduce the time taken to prepare coverslips for templating. To prepare channel templates using a PDMS mould, first a pattern was formed using a CO_2_ laser micromachining tool (VLS2.30, Universal Laser Systems) to cut a 1 mm thick acrylic sheet. PDMS was poured onto the laser cut acrylic. Once cured, PDMS was peeled from the acrylic, inverted and then silanised with tridecafluoro-1,1,2,2-tetrahydrooctyl-1-trichlorosilane (PFOCTS; Sigma, Europe). Uncured PDMS mixture was poured onto the silanised PDMS master and allowed to cure. Once cured this could be peeled and used to template electrodes.

### Micropatterning of electrodes

#### Preparation

For the preparation of the plating bath a modified Tollens’ reagent was used. Briefly, a silver nitrate solution (500 μL, 2 M) was reacted with 250 μL of ammonium hydroxide solution (28% v/v) to yield a solution, containing 1.33 M diamine silver (I). A separate solution of glucose (0.4 g/mL, 2.22 M), was also prepared. The silver solution was mixed with 500 μL of the glucose solution to yield a plating solution with a final silver species concentration of 0.8 M. 400 μL of the plating solution was added to each reservoir on top of each of the inlet holes. The chip was then placed in a desiccator and evacuated for 5 minutes. Upon introduction of atmospheric pressure, the solution filled all of the microfluidic channels with the plating solution. The chip was then placed on a pre-heated hot plate. The chips were removed from the hot plate once a continuous reflective-metallic network had been formed. Upon removal from the hot plate, the chips are cooled by rinsing with DI water, before disassembly of the PDMS chip. The electrodes are then rinsed with DI water so as to remove any residual salts still present and left to air dry. Electrically conductive silver paint (Maplin, UK) was used to form electrode pads in order to interface electronics to the microelectrodes. All conductivity measurements were made using the two-point probe method using a digital multimeter (TestSafe TSDM1). For the cell lysis experiments, polyethyleneimine (PEI, 1% w/v) was spun coat on microelectrodes at 2500 rpm. The electrodes were then allowed to dry overnight.

#### Characterisation

Electrodes were mechanically characterized via the scotch tape test in order to test whether they were sufficiently adhered to the glass substrate. Additionally, electrodes were characterized by cyclic voltammetry. A commercial portable potentiostat (Emstat 3, Palmsens) controlled by PSTrace software was employed for all voltammetric studies. The planar electrodes were also imaged by scanning electron microscopy; standard imaging was performed on a JEOL JSM 6010 LA (JEOL, Japan) and high-resolution imaging on a Zeiss Auriga Cross Beam. SEM images were obtained using 3 separate electrode devices to ensure reproducibility. Samples were mounted onto metal stubs with double sided carbon tape and gold-coated with an automated sputter coater (Emtech K575X, Quorum Technologies) for 10 min prior to imaging.

### Antibody printing

To assay protein released from electrically lysed cells in a microwell array, a GFP antibody microarray is printed onto PEI-coated electrode-patterned glass coverslipsby an OmniGrid Micro microarrayer (Digilab, UK) using 946MP2 pins (ArrayIt, USA). The printing solution was prepared by mixing at a 1:1 ratio the antibody solution to the printing solution, comprising 6X SSC, 3 M Betaine and 0.01% SDS in PBS. To ensure good alignment, the angle of the electrodes relative to the edges of the coverslip was first measured using an inverted microscope (10X objective, Nikon Ti-E; Nikon, Japan). The microarrayer software (Axsys software; Digilab, UK) was then modified to print a rectangular array onto the coverslip rotated at this angle.

### Cell Culture

BE cells (BE-GFP; ICR, UK), a human colon carcinoma cell line. Stably expressing cytoplasmic GFP were used in experiments. Cells were cultured in a 5% CO_2_ 37 °C incubator using high glucose Dulbecco’s Modified Eagles Medium (DMEM; Invitrogen, UK) supplemented with 10% (v/v) foetal bovine serum (FBS, Invitrogen, UK), 1x non-essential amino acids and 1x penicillin, streptomycin and glutamate solution. Cells were grown in 25 cm^2^ flasks and at 80% confluence were harvested for experiments using a trypsin/EDTA solution. To stain cell nuclei, prior to detachment cells are incubated with 300 nM Hoechst 33342 in media for 5 min and washed 3× with media to remove dye solution. Trypsin is inactivated by adding growth media. Cells are then pelleted by centrifugation for 5 min at 200 g and resuspended in 4% bovine serum albumen in PBS (PBSA). Cell concentration is determined using a haemocytometer and the volume was adjusted with 4% PBSA to achieve the desired cell concentration.

### Image Acquisition

Brightfield and fluorescence microscopy was performed on an inverted microscope (Nikon Ti-E; Nikon, Japan) using a mercury lamp (Nikon, Japan) using standard DAPI and FITC filter sets. Total internal reflection fluorescence (TIRF) microscopy is performed to assess the binding of GFP protein to the anti-GFP antibody spots in each microwell. For TIRF measurements, a laser served as the excitation source at 488 nm and aligned using a motorised TIRF illumination unit (Nikon, Japan). Images were acquired with an electron-multiplied CCD camera (IXON DU-897E; Andor Technologies, Ireland) and analysed using Fiji and Matlab.

## Electronic supplementary material

Supplementary Information

Supplementary Video S1
