# Supplementary material for: Micropatterning of planar metal electrodes by vacuum filling microfluidic channel geometries
Source: Sci Rep. 2018 Sep 26;8:14380. doi: 10.1038/s41598-018-32706-6 (PMC6158193; doi:10.1038/s41598-018-32706-6)
Supplement: Supplementary file 1 — Supplementary Information [file 41598_2018_32706_MOESM1_ESM.docx]

**Micropatterning of planar metal electrodes by vacuum filling microfluidic channel geometries**

S. Chatzimichail^1,2^, P. Supramaniam^1,2^, O. Ces^1,2,3^ and A. Salehi-Reyhani^1,2,3†^

^1^ Department of Chemistry, Molecular Sciences Research Hub, Imperial College London, London, W12 0BZ, UK.

^2^ Institute of Chemical Biology, Molecular Sciences Research Hub, Imperial College London, London, W12 0BZ, UK.

^3^ fabriCELL, Molecular Sciences Research Hub, Imperial College London, London, W12 0BZ, UK.

^†^Corresponding author [ali.salehi-reyhani@imperial.ac.uk](mailto:ali.salehi-reyhani@imperial.ac.uk)

**Supplementary Information**

| 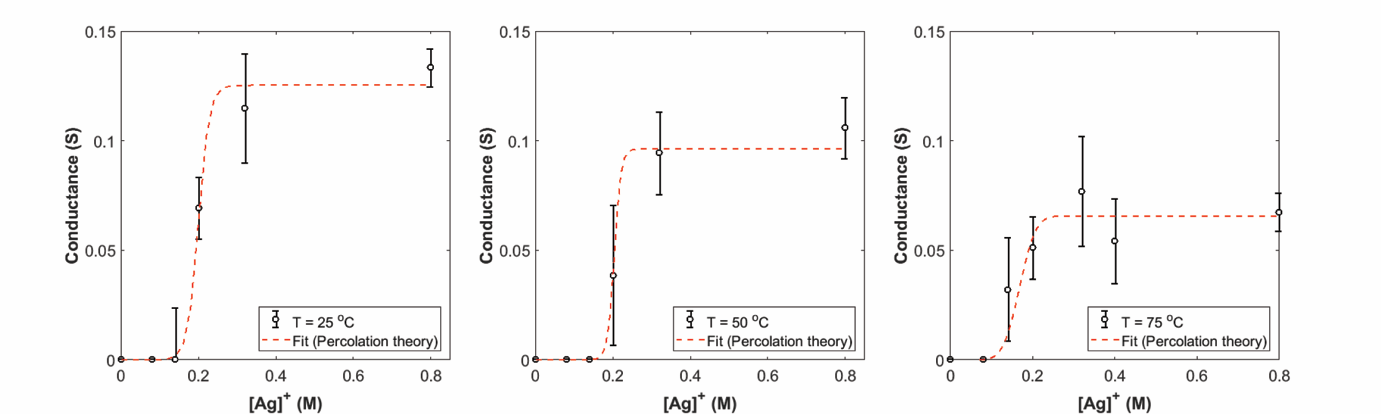 |
| --- |
| **Figure S1:** Producing microelectrodes. The conductance of the silver thin films was measured for a range of initial silver ion concentrations, [Ag]^+^, filling the microfluidic channels and hot plate temperatures. Error bars are the standard deviation of measurements made in triplicate. Dashed red lines are Boltzmann fits to the data suggesting that electrode formation is in line with percolation theory. |

| 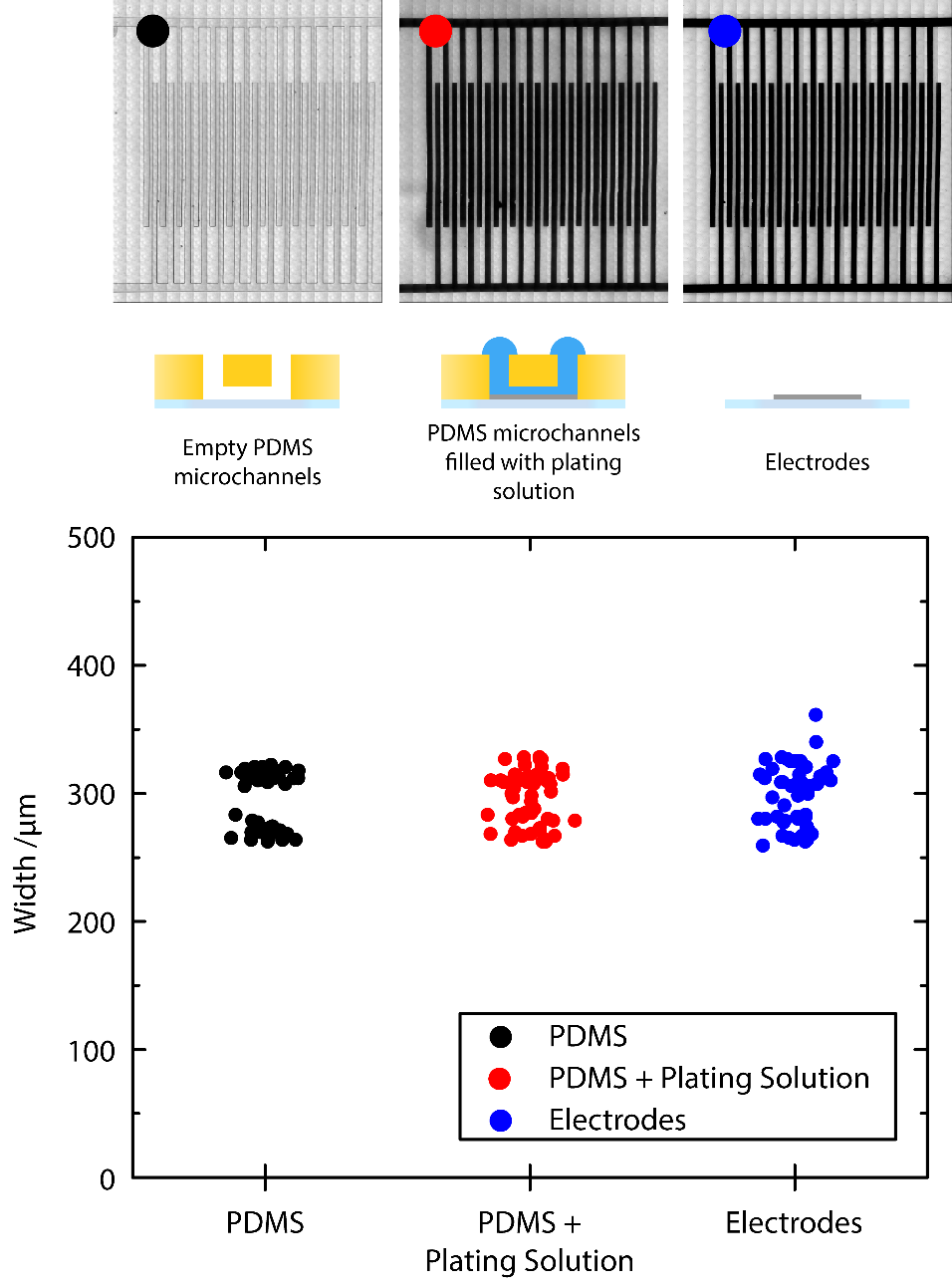 |
| --- |
| **Figure S2:** Characterising electrode geometry. To test whether the geometry of the PDMS microchannel was maintained to the final electrode, the widths were measured of (i) the empty PDMS microchannels, (ii) the PDMS microchannels vacuum filled with plating solution, and (iii) the bare microelectrodes that were produced (n = 56 electrodes). |

| 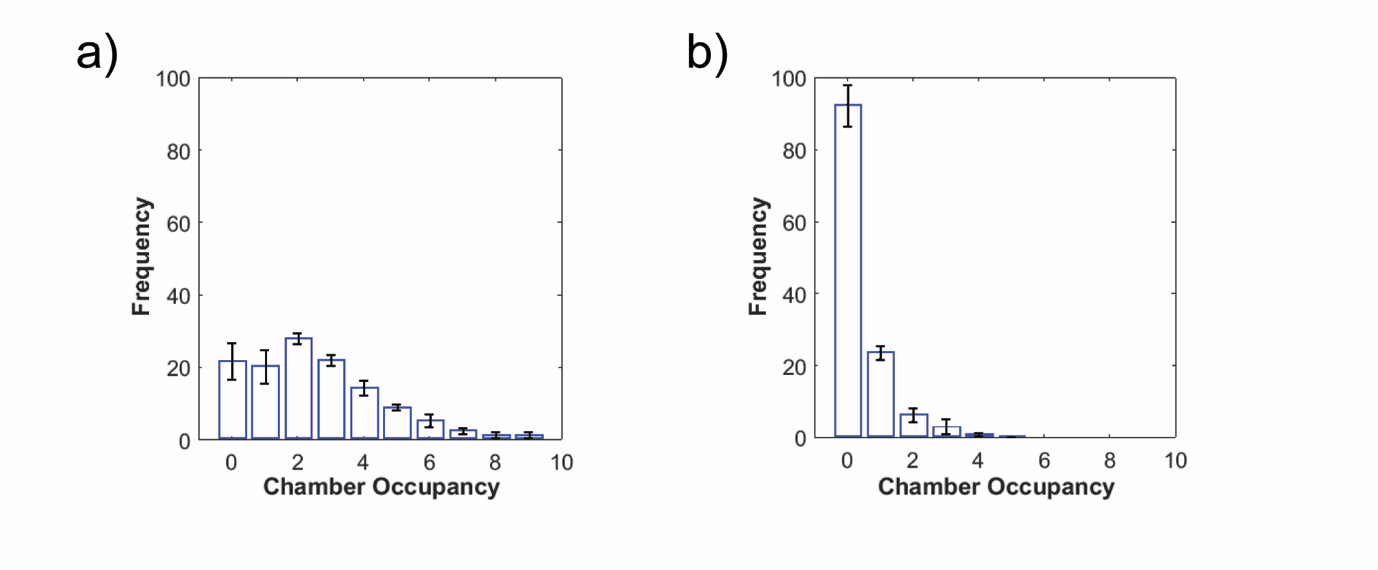 |
| --- |
| **Figure S3:** Optimising single cell occupancy of microwells. A suspension of single cells is allowed to sediment into the microwell array. Microwells are imaged and the number of cells per well are counted. Shown are histograms of the number of MCF7-GFP cells per microwell for solutions with cell concentrations of a) of 0.03 cells nL^-1^ and b) 0.33 cells nL^-1^. The single occupancy of the devices as determined in a triplicate of measurements was a) 24 ± 2% and b) 20 ± 5 %. |

**Supplementary Material – Video S1:** Lysis of a single cell in a microwell aligned to interdigitated electrode. Overlayed text indicates the time in seconds that the field to induce electrical lysis has been applied. The composite video is comprised of a brightfield channel (greyscale) and two fluorescence channels indicating GFP fluorescence (green) and a nuclear stain (blue). Field of view is 409.6 μm × 409.6 μm.
